# The pre-synaptic vesicle protein synaptotagmin is a novel biomarker for Alzheimer’s disease

**DOI:** 10.1186/s13195-016-0208-8

**Published:** 2016-10-03

**Authors:** Annika Öhrfelt, Ann Brinkmalm, Julien Dumurgier, Gunnar Brinkmalm, Oskar Hansson, Henrik Zetterberg, Elodie Bouaziz-Amar, Jacques Hugon, Claire Paquet, Kaj Blennow

**Affiliations:** 1 Department of Psychiatry and Neurochemistry, Institute of Neuroscience and Physiology, The Sahlgrenska Academy at the University of Gothenburg, Sahlgrenska University Hospital, Mölndal, SE-431 80 Sweden; 2 Clinical Neurochemistry Laboratory, Sahlgrenska University Hospital, Mölndal, Sweden; 3 Centre Mémoire de Ressources et de Recherche (CMRR) Paris Nord Ile de France, INSERM UMR-S942, Groupe Hospitalier Lariboisière Fernand-Widal Saint-Louis, Paris, France; 4 Clinical Memory Research Unit, Department of Clinical Sciences, Lund University, Malmö, Sweden; 5 Memory Clinic, Skåne University Hospital, Malmö, Sweden; 6 Department of Molecular Neuroscience, UCL Institute of Neurology, London, UK; 7 Service de Biochimie, Groupe Hospitalier Lariboisiere FW Saint-Louis, APHP, Université Paris Diderot, 75010 Paris, France

**Keywords:** Alzheimer’s disease, Biomarker, Cerebrospinal fluid, Synaptotagmin, Mass spectrometry, Immunopurification, Selected reaction monitoring, Parallel reaction monitoring

## Abstract

**Background:**

Synaptic degeneration is a central pathogenic event in Alzheimer’s disease that occurs early during the course of disease and correlates with cognitive symptoms. The pre-synaptic vesicle protein synaptotagmin-1 appears to be essential for the maintenance of an intact synaptic transmission and cognitive function. Synaptotagmin-1 in cerebrospinal fluid is a candidate Alzheimer biomarker for synaptic dysfunction that also may correlate with cognitive decline.

**Methods:**

In this study, a novel mass spectrometry-based assay for measurement of cerebrospinal fluid synaptotagmin-1 was developed, and was evaluated in two independent sample sets of patients and controls. Sample set I included cerebrospinal fluid samples from patients with dementia due to Alzheimer’s disease (*N* = 17, age 52–86 years), patients with mild cognitive impairment due to Alzheimer’s disease (*N* = 5, age 62–88 years), and controls (*N* = 17, age 41–82 years). Sample set II included cerebrospinal fluid samples from patients with dementia due to Alzheimer’s disease (*N* = 24, age 52–84 years), patients with mild cognitive impairment due to Alzheimer’s disease (*N* = 18, age 58–83 years), and controls (*N* = 36, age 43–80 years).

**Results:**

The reproducibility of the novel method showed coefficients of variation of the measured synaptotagmin-1 peptide 215–223 (VPYSELGGK) and peptide 238–245 (HDIIGEFK) of 14 % or below. In both investigated sample sets, the CSF levels of synaptotagmin-1 were significantly increased in patients with dementia due to Alzheimer’s disease (*P* ≤ 0.0001) and in patients with mild cognitive impairment due to Alzheimer’s disease (*P* < 0.001). In addition, in sample set I the synaptotagmin-1 level was significantly higher in patients with mild cognitive impairment due to Alzheimer’s disease compared with patients with dementia due to Alzheimer’s disease (*P* ≤ 0.05).

**Conclusions:**

Cerebrospinal fluid synaptotagmin-1 is a promising biomarker to monitor synaptic dysfunction and degeneration in Alzheimer’s disease that may be useful for clinical diagnosis, to monitor effect on synaptic integrity by novel drug candidates, and to explore pathophysiology directly in patients with Alzheimer’s disease.

**Electronic supplementary material:**

The online version of this article (doi:10.1186/s13195-016-0208-8) contains supplementary material, which is available to authorized users.

## Background

Alzheimer’s disease is characterized by intracellular neurofibrillary tangles, extracellular accumulation of aggregated amyloid-β, neuronal degeneration, and synaptic loss [1]. Several cerebrospinal fluid (CSF) biomarkers for Alzheimer’s disease are available, including total tau and phosphorylated tau protein, reflecting neurodegeneration and tau pathology, respectively, and amyloid-β_1–42_ reflecting deposition of the peptide into plaques [2, 3]. Numerous studies have consistently shown a marked increase in CSF total tau and phosphorylated tau accompanied by a reduction in amyloid-β_1–42_ in Alzheimer’s disease, and also in the mild cognitive impairment (MCI) stage of the disease [2, 3].

The leading hypothesis on Alzheimer’s disease pathogenesis poses that accumulation of amyloid-β in the brain is the primary driving force that causes synaptic failure and neurodegeneration, which leads to progressive cognitive deficits [4, 5]. The accumulation into amyloid-β may occur as early as 20–30 years before the first cognitive symptoms [6]. Synaptic degeneration in Alzheimer’s disease is related to cognitive decline [7–10] and synaptic loss occurs early in the disease [11, 12]. Therefore, synaptic biomarkers could be valuable tools for the disease, reflecting synaptic dysfunction, degeneration, or loss. In recent years, promising results have been published for some synaptic biomarkers in CSF, including the pre-synaptic protein synaptosomal-associated protein 25 (SNAP-25) [13] and the post-synaptic protein neurogranin [14] (http://www.alzforum.org/alzbiomarker/meta-analysis/alzheimers-disease-vs-control-neurogranin-csf). A marked increase of these synaptic CSF markers were found in dementia due to Alzheimer’s disease and in MCI due to Alzheimer’s disease, with higher CSF levels correlating with more marked future cognitive decline among MCI patients [13, 14].

Disruption of synaptic transmission may account for neuronal dysfunction and neurodegeneration. Synaptotagmin-1 is a pre-synaptic calcium sensor protein indispensable for synaptic vesicle exocytosis mediating neurotransmitter release in hippocampal neurons [15–18]. Efficient sustained neurotransmitter release is also dependent on reformation of synaptic vesicles after stimulation by endocytosis, where synaptotagmin-1 is functioning as an essential vesicle cargo molecule [18]. The crucial function of syntaptotagmin-1 in synaptic transmission [18–20] makes it a potential biomarker candidate reflecting synaptic dysfunction and degeneration in Alzheimer’s disease. Indeed, several studies have found a marked reduction of synaptotagmin-1 in typical disease-affected cortical brain regions in Alzheimer’s disease [21, 22] and a colocalization of synaptotagmin-1 with neuritic plaques [23]. We have previously shown that synaptotagmin is present in CSF [24], but until now there has not been any available assay for assessment of synaptotagmin in individual CSF samples.

In this study we report a novel mass spectrometry-based assay for measurement of the pre-synaptic protein synaptotagmin-1 in CSF. The aim of the study was to investigate the potential of synaptotagmin-1 as a CSF biomarker in dementia due to Alzheimer’s disease and in MCI due to Alzheimer’s disease. Because synaptic degeneration is a prominent feature in the brain in Alzheimer’s disease, we expected synaptotagmin-1 to be altered in these patients compared with controls and therefore it might serve as a valuable biomarker for Alzheimer’s disease.

## Methods

### CSF samples

The reproducibility of the analytical method was investigated in pooled left-over aliquots from decoded CSF samples supplied by the clinical routine section at the Clinical Neurochemistry Laboratory, The Sahlgrenska University Hospital, Mölndal, Sweden, following procedures approved by the Ethical Committee at University of Gothenburg. The quality control CSF pool 1 (QC1 sample) had an amyloid-β_1–42_ level of 586 ng/L and a total tau level below 75 ng/L. The QC2 sample had an amyloid-β_1–42_ level of 129 ng/L, a total tau level below 75 ng/L, and a phosphorylated tau level below 15 ng/L.

### Selection of the patients

CSF samples from subjects with either dementia due to Alzheimer’s disease or MCI due to Alzheimer’s disease or from a neurological control group were obtained from the Research Memory Center at Lariboisière Fernand-Widal University Hospital APHP. This department is highly experienced in the care management of patients with cognitive disorders and neurodegenerative disease, and has used CSF biomarkers for a long period of time [25–27]. Patients underwent a comprehensive clinical examination including personal medical and family histories, neurological examination, neuropsychological assessment, lumbar puncture with CSF biomarker analysis, and a brain structural imaging study with MRI. A consensus diagnosis was made by several clinicians (neurologists, geriatricians), neuropsychologists, and a biologist who are experts in CSF biomarkers. For each patient, diagnosis was made considering CSF results and according to validated clinical diagnostic criteria for dementia due to Alzheimer’s disease [28], MCI due to Alzheimer’s disease [29, 30], subjective cognitive impairment [31], and psychiatric disorder (DSM-IV). For all patients, diagnoses were validated in a second step by a neurologist (CP) and a biochemist (EB-A) before selecting CSF samples. In the absence of consensus diagnosis and in cases of disagreement about the final diagnosis, patients were not included in the study. According to this method, CSF from subjects with dementia due to Alzheimer’s disease, subjects with MCI due to Alzheimer’s disease, and neurological controls (no neurodegenerative disorders) was selected. Two sample sets were explored. Recently, the Alzheimer’s disease core CSF biomarkers have been included in the research criteria for the diagnosis of both early and manifest AD by the International Working Group [29] and in the diagnostic guidelines from the National Institute on Aging–Alzheimer’s Association [28], respectively. The following cutoff values were used to define a biochemical Alzheimer’s disease signature as supportive criteria for dementia due to Alzheimer’s disease [28]: amyloid-β_1–42_ (<550 ng/L), total tau (>400 ng/L), and phosphorylated tau (>50 ng/L). These criteria was also applied to exclude other diseases and to specify the controls. Sample set I included patients who underwent a lumbar puncture between 2010 and 2012, while sample set II included patients from 2013 to 2015 (Table 1).

### CSF collection

CSF was obtained by lumbar puncture between the L3/L4 or L4/L5 intervertebral space, using an atraumatic 24-gauge needle, collected in 10-mL polypropylene tubes. Samples were centrifuged at 1800 × *g* for 10 minutes at +4 °C, were aliquoted in 500 μL polypropylene tubes, and were stored at –80 °C pending analysis. Samples were frozen at −80 °C within 1 h after collection according to a standardized protocol described in a previous report [26]. A small amount of CSF was used for routine analysis, including total cell count, bacteriologic examination, and total protein and glucose levels.

### Analysis of CSF biomarkers

Amyloid-β_1–42_, total tau, and tau phosphorylated at threonine 181 (phosphorylated tau) protein measurements were performed using commercially available assays from Fujirebio (INNOTEST® β-AMYLOID(1-42), INNOTEST® hTAU Ag, and INNOTEST® PHOSPHO-TAU(181P)) according to the manufacturer’s instructions. For the two sample sets, the analysis of these biomarkers was performed in a single hospital laboratory (Lariboisère Hospital Paris) in two runs and averaged results were used for statistical analyses. The quality of CSF evaluations was validated by the Alzheimer’s Association quality control program for CSF biomarkers [32].

### Antibodies and recombinant protein of synaptotagmin-1

The monoclonal antibody clone 41.1 recognizing the cytoplasmic portion of synaptotagmin-1 was purchased from Synaptic Systems (Göttingen, Germany). Recombinant protein of synaptotagmin-1 (catalog number TP327252), transcript variant 3, was from OriGene Technologies, Inc. (Rockville, MD, USA).

### Immunoprecipitation

The immunoprecipitation method for CSF samples was performed according to Brinkmalm et al. [13] with minor modifications. Briefly, an aliquot (1 μg) of the mouse monoclonal synaptotagmin-1 antibody clone 41.1 (1 g/L) or IgG from murine serum (1 g/L, a negative control; Sigma-Aldrich) was separately added to 50 μL magnetic Dynabeads M-280 Sheep anti-mouse IgG (Invitrogen Corporation) and incubated for 1 h on a rocking platform at room temperature. The beads were washed three times with 1 ml of phosphate-buffered saline (PBS). The antibodies were cross-linked using 20 mM dimethyl pimelimidate dihydrochloride (Sigma-Aldrich) and 0.2 M triethanolamine (pH 8.2; Sigma-Aldrich) according to the manufacturer’s product description. The cross-linked beads were washed twice in PBS and were blocked with Roti-Block (Carl Roth) for 1 h on a rocking platform at room temperature. CSF samples (250 μL) were adjusted with 5 % Tween 20 and PBS to a final concentration of 0.05 % Tween 20 and a final volume of 1 mL. Samples and magnetic beads were incubated overnight on a rocking platform at +4 °C. The magnetic beads/sample solution was transferred to the KingFisher magnetic particle processor (Thermo Fisher Scientific), tube 1. The following three wash steps (tubes 2–4) were conducted for 10 s in 1 mL of each washing buffer: (tube 2) 0.025 % Tween 20 in PBS, (tube 3) PBS, and (tube 4) 50 mM ammonium hydrogen carbonate (NH_4_HCO_3_, pH 8.0). Synaptotagmin-1 was then eluted from the beads by adding 100 μL of 0.5 % formic acid (tube 5) for 4 min. The eluted fractions were transferred to 0.65 mL prelubricated Costar Microcentrifuge Tube (Fisher Scientific) and dried in a vacuum centrifuge.

### Protein digestion and addition of heavy-isotope-labeled peptide standards

Two isotopically labeled custom-made peptides containing U-13C6, U-15 N2-lysine[K] (aa 215–223, VPYSELGG[K] and aa 238–245, HDIIGEFK HeavyPeptide FasTrack 1 standards; Thermo Fisher Scientific) were dissolved in MilliQ water (~500 pmol/μL), mixed, and diluted in 50 mM NH_4_HCO_3_ to a final concentration of ~10 fmol/μL. The dried immunoprecipitated CSF samples were dissolved in 25 μL of a mixture of trypsin and labeled peptides (1 μg Sequencing Grade Modified Trypsin (Promega) dissolved in 0.01 % aqueous HCl (0.1 g/L) and diluted to 5 mg/L in isotopically labeled peptide mixture in 50 mM NH_4_HCO_3_, pH ≈ 7.8 (see earlier)), vortexed carefully, and incubated at +37 °C overnight. To stop the enzymatic activity, 5 μL of 10 % aqueous FA was added. The samples were centrifuged (16,900 × *g*, 10 min, +4 °C) and 27 μL of each sample was transferred to LC-vials (SUN-SRi).

### High-resolution parallel reaction monitoring analyses

High-resolution parallel reaction monitoring (HR-PRM) analyses were performed on a Q Exactive quadrupole–orbitrap mass spectrometer (Thermo Fisher Scientific) coupled to an Ultimate 3000 standard liquid chromatography system (Thermo Fisher Scientific). The samples (25 μL) were loaded directly onto a Hypersil GOLD HPLC C18 column (length 100 mm; inner diameter 2.1 mm; particle size 1.9 μm; Thermo Fisher Scientific) with 0.1 % aqueous formic acid at 100 μL/min. Mobile phases were: A, 0.1 % formic acid in water (v/v); and B, 0.1 % formic acid and 84 % acetonitrile in water (v/v). The peptides were eluted off the column using the following linear gradient steps: 1 min, 0 % B; 3 min, 12 % B; 11 min, 40 % B; and 13 min, 100 % B. The IonMax electrospray ion source settings were: spray voltage, +4100 V; capillary temperature, +320 °C; sheath gas pressure setting, 25 arbitrary units; auxiliary gas pressure setting, 10 arbitrary units; and heater temperature, +300 °C. The instrument was set to acquire scheduled pairs of SIM scans and subsequently all ion fragmentation scans (isolation window 8.0 *m/z*) in profile mode, allowing simultaneous detection of both the synaptotagmin peptide and the corresponding isotopically labeled peptide standard. The settings were common for both scan types and were as follows: resolution setting, 35,000; AGC target, 1e6; maximum injection time, 120 ms. Data acquisition and analysis were performed with Xcalibar software version 2.2 SP1.48 (Thermo Fisher Scientific).

### Assay reproducibility

The intra-day variation of synaptotagmin was determined using two QC samples (QC1 and QC2). The immunoprecipitation of synaptotagmin-1 from the QC samples were performed on different days but analyzed on the same occasion. Assessment of reproducibility was performed on two different occasions, and QC samples were randomized in between samples from sample set I (first occasion) and sample set II (second occasion).

### Statistical analysis

Because most of the analytes were not normally distributed (Shapiro–Wilk test, *P* < 0.05), nonparametric statistics were used for analysis. Data are given as the median (interquartile range). Differences between more than two groups were assessed with Kruskal–Wallis test. Statistically significant results (*P* < 0.05) were followed by Mann–Whitney U tests to investigate group differences. Receiver operating characteristic (ROC) curves were performed on each subject group on the tryptic peptides of synaptotagmin-1 in order to assess their diagnostic value. For each tryptic peptide of synaptotagmin, the area under the curve and a 95 % confidence interval was calculated using GraphPad Prism 5. The correlation coefficients (rho) were calculated using the Spearman two-tailed correlation test. SPSS 20.0 was employed for most of the statistical analyses.

## Results

### Assay performance

The reproducibility of the novel method showed coefficients of variation (CV) of the measured synaptotagmin-1 peptide 215–223 (VPYSELGGK) and peptide 238–245 (HDIIGEFK) of 14 % or below (Additional file 1: Table S1). The investigated tryptic peptides correlated with each other in the groups of controls (rho = 0.971, *P* < 0.00001, sample set I; and rho = 0.995, *P* < 0.00001, sample set II), MCI due to Alzheimer’s disease (rho = 0.988, *P* < 0.00001, sample set II; sample set I was too small for statistical evaluation), and dementia due to Alzheimer’s disease (rho = 0.980, *P* < 0.00001, sample set I; and rho = 0.995, *P* < 0.00001, sample set II) (Table 2).

### Clinical studies

#### Demographics

Table 1 presents the demographic characteristics of the groups. Sample set I consisted of five patients with MCI due to Alzheimer’s disease (one man and four women, age 62–88 years), 17 patients with dementia due to Alzheimer’s disease (five men and 12 women, age 52–86 years), and 17 neurological controls (seven men and 10 women, age 41–82 years). The replication sample set (sample set II) consisted of 18 patients with MCI due to Alzheimer’s disease (five men and 13 women, age 58–83 years), 24 patients with dementia due to Alzheimer’s disease (seven men and 17 females, age 52–84 years), and 36 neurological controls (13 men and 23 women, age 43–80 years). In sample set I, the patients with MCI due to Alzheimer’s disease were older than the subjects with controls. Both patients with MCI due to Alzheimer’s disease and patients with dementia due to Alzheimer’s disease were significantly older than the subjects with controls in sample set II. All patients from the neurological control group had cognitive complaints (group I, *N* = 14 and group II, *N* = 25) or had psychiatric diagnoses (group I, *N* = 3 and group II, *N* = 11). To rule out preclinical Alzheimer’s disease in the neurological control group, only cases with normal CSF levels amyloid-β_1–42_ (>550 ng/L), total tau (<400 ng/L), and phosphorylated tau (<50 ng/L) were included.

**Table 1:** Demographic data and biomarker CSF levels for the diagnostic groups^a^

	Control	MCI-AD	Alzheimer’s disease dementia
Sample set I			
Number (men/women)	17 (7/10)	5 (1/4)	17 (5/12)
Age (years)	60 (53–67), *P* = 0.02^c^	78 (73–81), *P* = 0.02^b^	65 (58–81)
MMSE	27 (24–28)	27 (27–28)	21 (16–23), *P =* 0.00001^b^, *P* = 0.004^c^
Amyloid-β_1–42_ (ng/L)	838 (697–998), *P* = 0.001^c^	539 (316–582), *P =* 0.001^b^	398 (319–483), *P* < 0.00001^b^
Total tau (ng/L)	180 (126–206), *P* = 0.00008^c^	1000 (766–1078), *P* = 0.00008^b^	602 (442–769), *P <* 0.00001^b^
Phosphorylated tau (ng/L)	39 (35–42), *P* = 0.00008^c^	143 (97–180), *P* = 0.00008^b^	89 (75–119), *P* < 0.00001^b^
Sample set II			
Number (men/women)	36 (13/23)	18 (5/13)	24 (7/17)
Age (years)	62 (55–69), *P* = 0.001^c^	70 (69–78), *P* = 0.001^b^	68 (64–72), *P* = 0.02^b^
MMSE	28 (26–29)	27 (26–28)	22 (17–24), *P =* 0.00001^b^, *P* = 0.00001^c^
Amyloid-β_1–42_ (ng/L)	971 (844–1110), *P* < 0.00001^c^	558 (377–667), *P* < 0.00001^b^	497 (459–571), *P* < 0.00001^b^
Total tau (ng/L)	197 (157–227), *P* < 0.00001^c^	570 (520–717), *P* < 0.00001^b^	623 (527–799), *P* < 0.00001^b^
Phosphorylated tau (ng/L)	40 (32–46), *P* < 0.00001^c^	86 (79–104), *P* < 0.00001^b^	91 (73–115), *P* < 0.00001^b^

^a^Data given as median (interquartile range) unless otherwise indicated. Statistical differences were determined using nonparametric tests

^b^Compared with controls

^c^Compared with MCI-AD

*MCI-AD* mild cognitive impairment due to Alzheimer’s disease, *MMSE* Mini-Mental State Examination

**Fig. 1:**
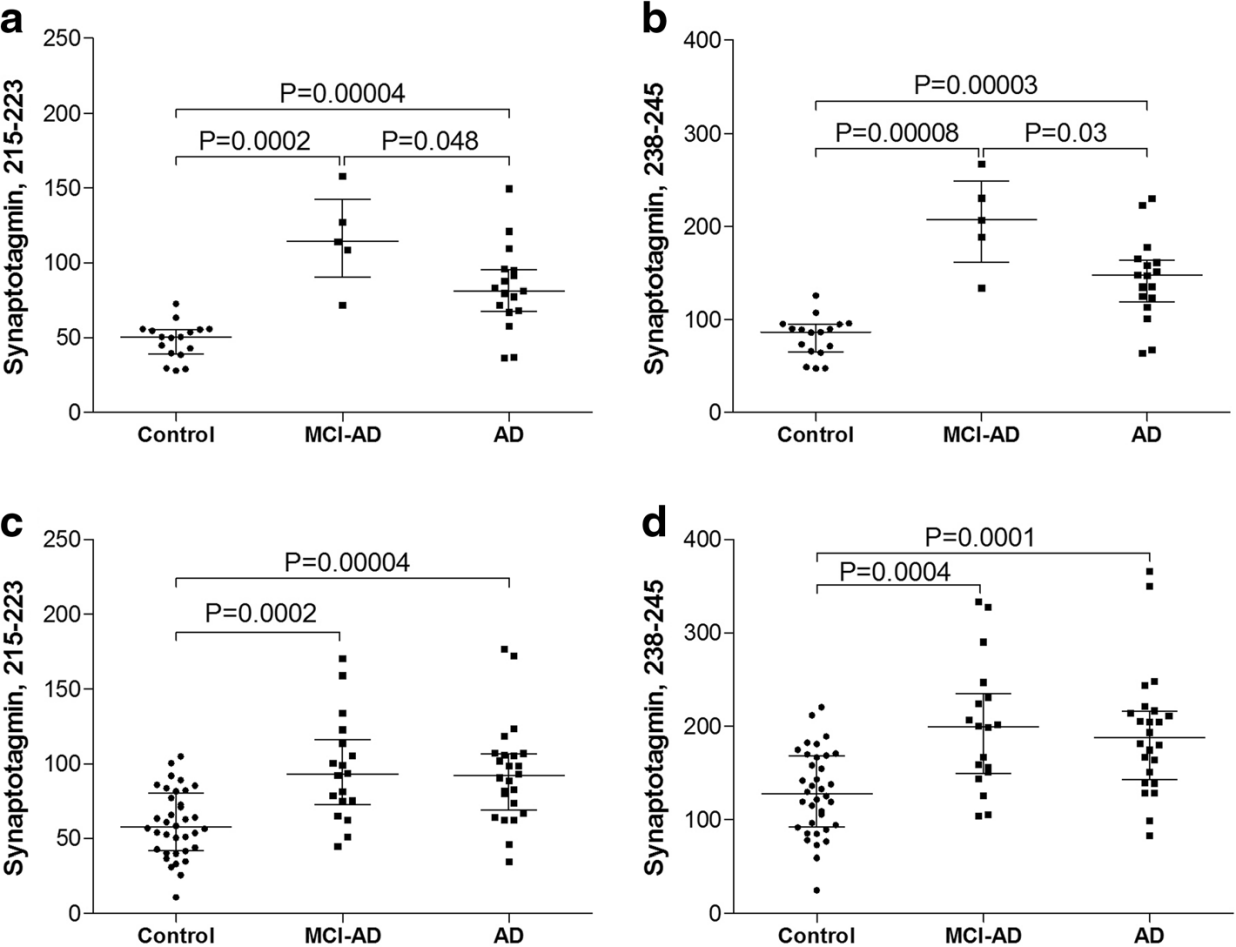
Targeted HR-PRM-MS analyses of synaptotagmin in human CSF. Individual values for the HR-PRM-MS measured peak area ratios ((endogenous peptide/labeled peptide standard) × 1000) of immunoprecipitated synaptotagmin in CSF samples within sample set I (**a**, **b**) and sample set II (**c**, **d**). The four panels depict the measured levels of two tryptic peptides of synaptotagmin, 215–223 (VPYSELGGK) (**a**, **c**) and 238–245 (HDIIGEFK) (**b**, **d**). *AD* dementia due to Alzheimer’s disease, *MCI-AD* mild cognitive impairment due to Alzheimer’s disease

The CSF levels of the investigated tryptic peptides of synaptotagmin-1 were significantly higher in patients with MCI due to Alzheimer’s disease and patients with dementia due to Alzheimer’s disease compared with controls (Fig. 1). In sample set I, the tryptic peptides of synaptotagmin-1 were significantly higher in patients with MCI due to Alzheimer’s disease compared with patients with dementia due to Alzheimer’s disease (Fig. 1a, b).

**Fig. 2:**
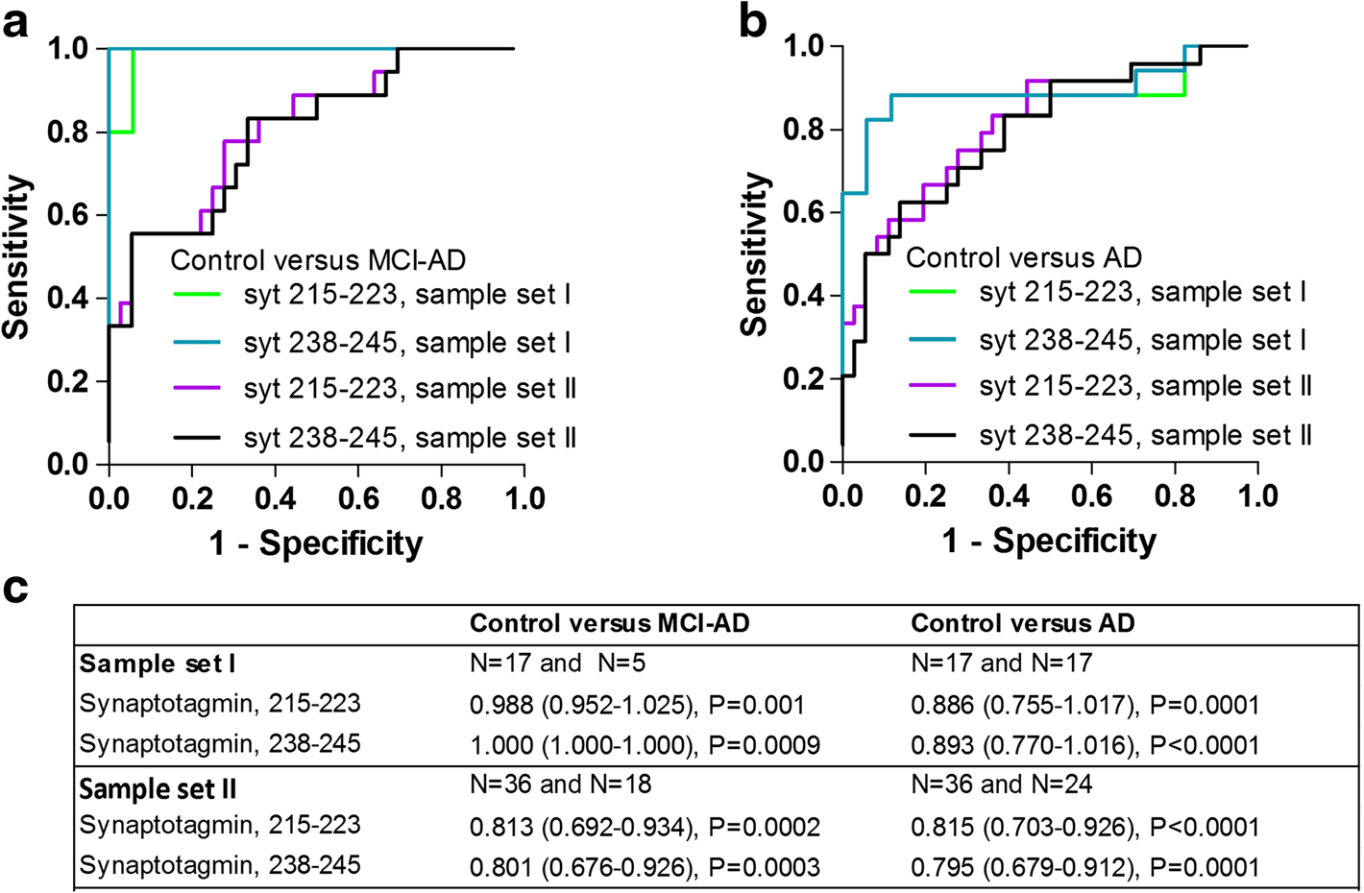
ROC curve analysis of synaptotagmin in human CSF. ROC curve analysis for synaptotagmin 215–223 (*green* and *pink*) and synaptotagmin 238–245 (*turquoise* and *black*) in CSF for differentiation of MCI due to Alzheimer’s disease (MCI-AD) from controls in sample set I and sample set II, respectively (**a**). ROC curve analysis for synaptotagmin 215–223 (*green* and *pink*) and synaptotagmin 238–245 (*turquoise* and *black*) in CSF for differentiation of dementia due to Alzheimer’s disease (AD) from controls in sample set I and sample set II, respectively (**b**). The area under the curve (95 % confidence interval) is shown in the included table (**c**). *AD* dementia due to Alzheimer’s disease, *MCI-AD* mild cognitive impairment due to Alzheimer’s disease (Color figure online)

Each of the tryptic peptide assays of synaptotagmin-1 (215–223 and 238–245) could differentiate MCI due to Alzheimer’s disease from controls in both sample sets, with area under the curve of 0.988 (0.952–1.025) (*P* = 0.001) and 1.000 (1.000–1.000) (*P* = 0.0009) (sample set I), respectively, and of 0.813 (0.692–0.934) (P = 0.0002) and 0.801 (0.676–0.926) (*P* = 0.0003) (sample set II), respectively (Fig. 2a). The tryptic peptide assays of synaptotagmin-1 (215–223 and 238–245) could also each differentiate dementia due to Alzheimer’s disease from controls in both sample sets, with area under the curve of 0.886 (0.755–1.017) (*P* = 0.0001) and 0.893 (0.770–1.016) (*P* < 0.0001) (sample set I), respectively, and of 0.815 (0.703–0.926) (*P* < 0.0001) and 0.795 (0.679–0.912) (*P* = 0.0001) (sample set II), respectively (Fig. 2b).

No statistically significant correlations between age and the levels of the tryptic peptides of synaptotagmin were observed in any of the investigated groups (Table 2). There were no statistical significant correlations between CSF synaptotagmin-1 and Mini-Mental State Examination (MMSE) scores in any group.

**Table 2:** Correlation between age, MMSE, and biomarker levels for the diagnostic groups^a^

	Synaptotagmin, 215–223	Synaptotagmin, 238–245
Sample set I, control (*N* = 17)		
Age	N.S.	N.S.
MMSE	N.S.	N.S.
Amyloid-β_1–42_ (ng/L)	rho = 0.515, *P* = 0.04	N.S.
Total tau (ng/L)	rho = 0.739, *P* = 0.001	rho = 0.749, *P* = 0.001
Phosphorylated tau (ng/L)	rho = 0.770, *P* = 0003	rho = 0.708, *P* = 0.001
Synaptotagmin, 215–223	–	rho = 0.971, *P* < 0.00001
Synaptotagmin, 238–245	rho = 0.971, *P* < 0.00001	–
Alzheimer’s disease dementia (*N* = 17)		
Age	N.S.	N.S.
MMSE	N.S.	N.S.
Amyloid-β_1–42_ (ng/L)	N.S.	N.S.
Total tau (ng/L)	rho = 0.540, *P* = 0.03	rho = 0.610, *P* = 0.009
Phosphorylated tau (ng/L)	rho = 0.586, *P* = 0.01	rho = 0.656, *P* = 0.004
Synaptotagmin, 215–223	–	rho = 0.980, *P* < 0.00001
Synaptotagmin, 238–245	rho = 0.980, *P* < 0.00001	–
Sample set II, control (*N* = 36)		
Age	N.S.	N.S.
MMSE	N.S.	N.S.
Amyloid-β_1–42_ (ng/L)	rho = 0.381, *P* = 0.02	rho = 0.348, *P* = 0.04
Total tau (ng/L)	rho = 0.641, *P* = 0.00003	rho = 0.633, *P* = 0.00004
Phosphorylated tau (ng/L)	rho = 0.687, *P* < 0.00001	rho = 0.683, *P* < 0.00001
Synaptotagmin, 215–223	–	rho = 0.995, *P* < 0.00001
Synaptotagmin, 238–245	rho = 0.995, *P* < 0.00001	–
MCI-AD (N = 18)		
Age	N.S.	N.S.
MMSE	N.S.	N.S.
Amyloid-β_1–42_ (ng/L)	N.S.	N.S.
Total tau (ng/L)	N.S.	N.S.
Phosphorylated tau (ng/L)	N.S.	N.S.
Synaptotagmin, 215–223	–	rho = 0.988, *P* < 0.00001
Synaptotagmin, 238–245	rho = 0.988, *P* < 0.00001	–
Alzheimer’s disease dementia (*N* = 24)		
Age	N.S.	N.S.
MMSE	N.S.	N.S.
Amyloid-β_1–42_ (ng/L)	N.S.	N.S.
Total tau (ng/L)	rho = 0.655, *P* = 0.001	rho = 0.675, *P* = 0.0003
Phosphorylated tau (ng/L)	rho = 0.653, *P* = 001	rho = 0.673, *P* = 0.0003
Synaptotagmin, 215–223	–	rho = 0.995, *P* < 0.00001
Synaptotagmin, 238–245	rho = 0.995, *P* < 0.00001	–

^a^Correlations presented by the Spearman’s rank correlation coefficient (rho). Nonsignificant (*N.S*., *P* > 0.05) correlations were not reported

*MCI-AD* mild cognitive impairment due to Alzheimer’s disease, *MMSE* Mini-Mental State Examination

The CSF levels of synaptotagmin (tryptic peptides 215–223 and 238–245) correlated with the levels of total tau and phosphorylated tau in both the control group (sample sets I and II) and in patients with dementia due to Alzheimer’s disease (sample sets I and II), but not in patients with MCI due to Alzheimer’s disease (sample set II) (Table 2). Synaptotagmin-1 (215–223) correlated with amyloid-β_1__-__42_ in the control group (rho = 0.515, *P* = 0.04 and rho = 0.381, *P* = 0.02, respectively), but not in patients with either MCI due to Alzheimer’s disease or dementia due to Alzheimer’s disease (Table 2). Synaptotagmin-1 (238–245) correlated only with amyloid-β_1__-__42_ (rho = 0.348, P = 0.04) in the control group of sample set II, while there were no correlations within other investigated groups (Table 2).

## Discussion

In this study, we investigated the potential of synaptotagmin-1 as a CSF biomarker in dementia due to Alzheimer’s disease and in MCI due to Alzheimer’s disease. Therefore, we developed novel assays for the measurement of synaptotagmin-1, which is a pre-synaptic vesicle protein indispensable for the regulation of synaptic transmission and for sustaining intact cognitive function. In the present study, we report that the levels of synaptotagmin-1 were significantly increased in patients with MCI due to Alzheimer’s disease and patients with dementia due to Alzheimer’s disease compared with controls, supporting that synaptotagmin-1 could be an early marker for Alzheimer’s disease.

The novel assay combines immunoprecipitation, tryptic digestion and finally mass spectrometry (HR-PRM) for analyses of the CSF levels of synaptotagmin, which is a similar approach to that we applied successfully for quantification of SNAP-25 in CSF [13]. Synaptotagmin-1 consists of an N-terminal intravesicular domain, a transmembrane part, and a large cytoplasmic sequence that contains two calcium (C2) binding domains. Both tryptic peptides of the novel assays are located in the first (C2A) of two calcium-binding domains of synaptotagmin-1, domains of the protein that are essential for appropriate synaptic transmission [16–18]. Furthermore, the novel mass spectrometry-based method allows selective measurements of the synaptotagmin-1 isoform. Since the 17 members of the synaptotagmin family are differently expressed throughout the body [33, 34] and these isoforms exhibit diverse biological functions, the ability to discriminate between them is certainly of great importance for a valuable synaptic biomarker.

We found that the CSF levels of synaptotagmin-1 were consistently elevated in patients with dementia due to Alzheimer’s disease compared with controls in two separate sample sets. Additionally, synaptotagmin-1 was increased already in MCI due to Alzheimer’s disease, supporting the notion that this synaptic marker might be an early marker for Alzheimer’s disease. These findings are in accordance with our previous findings of the pre-synaptic marker SNAP-25 [13] and the post-synaptic marker neurogranin [14]. Furthermore, synaptotagmin-1 was even higher in MCI due to Alzheimer’s disease compared with dementia due to Alzheimer’s disease, suggesting that synaptic dysfunction and degeneration can be identified before the onset of clinical dementia [11, 12]. However, because this latter finding was only supported by data from sample set I, this notion needs to be taken with caution and needs to be reproduced in a larger set of patients.

Each measured tryptic peptide of synaptotagmin-1 (215–223 and 238–245) could differentiate dementia due to Alzheimer’s disease from controls and MCI due to Alzheimer’s disease from controls to a similar magnitude, in the respective sample sets. The tryptic peptides of synaptotagmin-1 assays also correlated with each other in all investigated groups (controls, MCI due to Alzheimer’s disease, and dementia due to Alzheimer’s disease) in the respective sample sets. Moreover, the reproducibility of the novel method (CV) was less than 14 %. Altogether these findings support the notion that each tryptic peptide corresponds to the same synaptotagmin-1 molecule.

In both sample sets, the MCI due to Alzheimer’s disease patients were significantly older than the controls. In sample set I the patients with dementia due to Alzheimer’s disease and the controls were age matched, while the patients with dementia due to Alzheimer’s disease were significantly older than the controls in sample set II. On the other hand, there were no statistically significant correlations between age and the levels of synaptotagmin-1 in any of the investigated groups, suggesting that the CSF synaptotagmin-1 levels not are affected by age within the age range of this study.

The mechanism for release of synaptotagmin-1 into CSF is not known. However, it is interesting to note that previous studies showed that C2 domains may penetrate a cell membrane upon calcium binding [35, 36], which could be a possible mechanism of liberation into CSF. Herein, we found that synaptotagmin-1 correlated with the levels of total tau and phosphorylated tau both in the control group and in patients with dementia due to Alzheimer’s disease in both investigated sample sets. CSF total tau has previously been suggested to be a general marker of damage to cortical nonmyelinated neurons [2]. In contrast, phosphorylated tau might be a more specific marker for Alzheimer’s disease [2], since high CSF levels of phosphorylated tau have been found to correlate with the accumulation of cortical neurofibrillary tangles [37, 38]. To summarize, our results suggest that synaptotagmin-1 might be a specific marker for dementia due to Alzheimer’s disease that to some extent also might reflect general neurodegeneration.

The fact that synaptic loss is the main pathological feature of dementia due to Alzheimer’s disease that correlates with cognitive decline, together with the notion that synaptotagmin-1 is directly involved in the regulation of neurotransmitter release [16, 18], make synaptotagmin-1 a potential CSF biomarker to follow progression of clinical symptoms. In the present study, there were no correlations between cognitive decline and the tryptic peptides of synaptotagmin-1 in any of the examined groups, possibly due to the small size of the clinical sample sets. Further studies are therefore needed to investigate whether synaptotagmin-1 in CSF could be used for assessment of the future rate of cognitive decline.

The strength of the present study is that we present robust assays for assessment of the CSF levels of synaptotagmin-1 in two independent sample sets. To our knowledge this is the first study investigating the potential of synaptotagmin-1 as a CSF biomarker for Alzheimer’s disease. One limitation of the present study is the cross-sectional design of the clinical study that complicates the investigation of possible association between CSF synaptotagmin and synaptic degeneration. This question has to be addressed by longitudinal measurements of synaptotagmin to investigate whether the levels are changed over time and whether it correlates with cognitive alterations in patients with Alzheimer’s disease. Another limitation could probably be that all clinical samples were analyzed as single samples. Since the analytical reproducibility of the method (CV < 14 %) yields very similar results for duplicates while the measured levels vary considerably between patients, we decided to include as many different patients as possible and thereby try to obtain a more representative picture of the biological variation rather than the analytical.

## Conclusions

We present a novel method for measurement of the pre-synaptic protein synaptotagmin-1 in CSF samples. Synaptotamin-1 concentrations were increased in patients with MCI due to Alzheimer’s disease and patients with dementia due to Alzheimer’s disease compared with controls, supporting the notion that synaptotagmin-1 could be a valuable differential biomarker both in early Alzheimer’s disease and after manifestation of the disease.

## Additional file

Additional file 1: Table S1. Presenting the intra-assay varibility for the QC samples. (DOCX 46 kb)

## Abbreviations

CV: Coefficients of variation
CSF: Cerebrospinal fluid
MCI: Mild cognitive impairment
MMSE: Mini-Mental State Examination
PBS: Phosphate-buffered saline
SNAP-25: Synaptosomal-associated protein 25
ROC: Receiver operating characteristic
