# Supplementary material for: The pre-synaptic vesicle protein synaptotagmin is a novel biomarker for Alzheimer’s disease
Source: Alzheimers Res Ther. 2016 Oct 3;8:41. doi: 10.1186/s13195-016-0208-8 (PMC5048479; doi:10.1186/s13195-016-0208-8)
Supplement: Supplementary file 1 [file 13195_2016_208_MOESM1_ESM.docx]

Supplementary Table 1

The intra-assay varibility for the QC samples

Analysed at	Sample		synaptotagmin (aa 215-223)	synaptotagmin (aa 238-245)
The first occasion	CV (QC1)	IP performed at the same occasion.	4.4% (N=4)	4,4% (N=4)
	CV (QC1)	IP performed at the same occasion.	11% (N=4)	11% (N=4)
	CV (QC1)	IP performed at two different occasions.	8.7% (N=8)	10% (N=8)
	CV (QC2)	IP performed at the same occasion.	9.4% (N=3)	7.4% (N=3)
	CV (QC2)	IP performed at the same occasion.	9.1% (N=3)	13% (N=3)
	CV (QC2)	IP performed at two different occasions.	8.3% (N=6)	9.7% (N=6)
The second occasion	CV (QC1)	IP performed at the same occasion.	7,7% (N=4)	6,8% (N=4)
	CV (QC1)	IP performed at the same occasion.	13,5% (N=4)	0,13% (N=4)
	CV (QC1)	IP performed at the same occasion.	13% (N=4)	14% (N=4)
	CV (QC1)	IP performed at three different occasions.	11% (N=8)	12% (N=8)

Abbreviations: coefficient of variation (CV); immunoprecipitation (IP); Quality control (QC)
